# Phytochemical screening and protective effects of *Trifolium alexandrinum* (L.) against free radical-induced stress in rats

**DOI:** 10.1002/fsn3.152

**Published:** 2014-09-19

**Authors:** Abdus S Shah, Mushtaq Ahmed, Huda M Alkreathy, Muhammad R Khan, Rahmat A Khan, Samiullah Khan

**Affiliations:** 1 Department of Biotechnology, University of Science and TechnologyBannu, KPK, Pakistan; 2 Department of Pharmacology, Faculty of Medicine, King Abdulaziz UniversityJeddah, Saudia Arabia; 3 Department of Biochemistry, Faculty of Biological Sciences, Quaid-i-Azam UniversityIslamabad, Pakistan; 4 Department of Microbiology, Quaid-i-Azam UniversityIslamabad, Pakistan

**Keywords:** CCl_4_, kidney, lipid peroxidation, oxidative stress, *Trifolium alexandrinum*

## Abstract

*Trifolium alexandrinum* is traditionally used in various human ailments, including renal dysfunctions. The present experiment was designed to investigate antioxidant and nephroprotective effect of *T. alexandrinum* methanolic extract (TAME) against CCl_4_-induced oxidative stress in albino rats. Results of in vitro study revealed significant (*P* < 0.05) antioxidant effects. The ameliorative role of TAME was also examined by investigating the level of antioxidant enzymes catalase (CAT), peroxidase (POD), glutathione peroxidase (GSH-Px), glutathione-*S*-transferase (GST), nonenzymatic antioxidant viz; reduced glutathione contents (GSH) and lipid peroxidation products (TBARS) in the renal tissue homogenate in CCl_4_-treated rats. The intraperitoneal injection of 1 mL/kg b.w. CCl_4_ caused a significant depletion in the activity antioxidant enzymes and increased the TBARS contents. Supplementation of TAME at 200 mg/kg b.w. for 2 weeks significantly improved activities of antioxidant enzymes and reduced TBARS formation. Co-treatment of TAME also presented significant protection in maintaining renal urine and serum markers. Antioxidant and nephroprotective effects of TAME are associated with its polyphenolic constituents.

## Introduction

For the maintenance of normal physiology, the living cell constantly generates reactive oxygen species (ROS), and keeps them in a balanced state by the antioxidant defense system. The overproduction of ROS, beyond the range of antioxidant defense system, in the cell causes oxidative stress that damages macromolecules like DNA, protein, and lipids (Poulson et al. 1988). In vivo antioxidant defense system consists of antioxidant enzymes like SOD (superoxide dismutase), CAT (catalase) and GPx (glutathione peroxidase), and nutritional antioxidants. Disturbance in the balance of the oxidant–antioxidant system causes numerous diseases such as cancer, atherosclerosis, diabetes and degenerative diseases (Govindarajan et al. 2005). Certain environmental factors such as radiation, smoking, exposure to heavy metals and chemicals increased the normal production of ROS and induced oxidative stress (Kim et al. 1990). CCl_4_ causes nephrotoxicity in workers exposed to it as well as in experimental animals (Abraham et al. 1999). Inhalation, skin absorption, and ingestion of CCl_4_ increases lipid peroxidation and decreases protein content and the activity of antioxidant enzymes (Dashti et al. 1989; Daniels et al. 1995; Khan et al. 2009). CCl_4_ intoxication generates free radicals in kidney, lungs, heart, and blood cells. CCl_4_ is converted by cytochrome P450 system into very reactive trichloromethyle radicals (CCl_3_•) which in the aerobic condition form very toxic trichloromethyl peroxyl radicals (CCl_3_O_2_•) (Behar-Cohen et al. 1996; Khan et al. 2010a). The trichloromethyl free radicals combine with SH groups of glutathione (GSH) and proteins and initiate lipid peroxidation that leads toward cellular necrosis (Brautbar and Williams 2002; Adewole et al. 2007). Beside the antioxidant enzymes CCl_4_ also change the renal profile such as serum creatinine, albumins, and bilirubins (Khan et al. 2010b). Numerous reports have revealed that antioxidant therapy recover oxidative stress and lipid peroxidation. It is found that leaves of *Morus indica* have antioxidant properties in diabetic rats (Andallu and Varadacharyulu 2003). Medicinal plants possess bioactive polyphenolic constituents which scavenge reactive oxygen radicals and control oxidative stress (Mukherjee and Wahile 2006). Recently numerous medicinal plants like *Launaea procumbens* and *Euphorbia prostrata* have been screened and reported with proved antioxidant potential (Sun et al. 2002; Ahmad et al. 2006, 2011).

*Trifolium alexandrinum* (L) is a winter fodder and distributed all over the world and traditionally used in the treatment of various human dysfunctions and renal pain. The phytochemistry of this plant has indicated the presence of terpenoinds, flavonoids, isoflavonoids, and fatty acids (Sharaf 2008; Temine and Guler 2009). *Trifolium alexandrinum* (L) is reported as to be an antibacterial and antidiabetic agent (Khan et al. 2012). There is no pharmacological evidence in favor of its traditional uses. Therefore, the present study was arranged to investigate the antioxidant and renoprotective effects of *T. alexandrinum* (L) against CC1_4_-induced oxidative stress in rats.

## Material and Methods

### Chemicals

Glutathione in the reduced form, Glutathion-*S*-transferase, Glutathione oxidized, nicotinamide adenine dinucleotide phosphate (NADPH)-Tetra salt, Ethylene diamine tetra acetic acid (EDTA) disodium salt and Silymarin from Sigma–Aldrich, Germany. 1-chloro-2,4-dinitrobenzene (CDNB) from MERCK Schuchardt, Germany; thiobarbituric acid (TBA), tri chloro acetic acid and all other chemicals and reagents used were in the highest available pure form.

### Phytochemical analysis

Phytochemical analysis of various bioactive constituents viz; alkaloids (Harborne 1973), coumarins, cardiac glycosides, anthraquinones (Trease and Evans 1989), tannins, and flavonoids using standard protocols (Sofowara 1993).

### DPPH radical scavenging activity assay

The DPPH (1, 1-diphenyl-2-picryl-hydrazyl) assay was done according to the method of Brand-Williams et al. (1995) with some modifications. The scavenging activity was estimated based on the percentage of the DPPH radical scavenged as in the following equation:

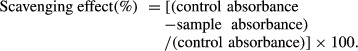


EC_50_ value is the effective concentration that could scavenge 50% of the DPPH radicals. Ascorbic acid was used as a positive reference.

### Animals

Twenty four-male Albino Wistar rats (140–220 g) were used in this study. Rats were provided by the Islamic international dental college Islamabad and fed with a standard laboratory diet and drinking water. The study protocol was approved by the Ethics Committee of animal care and use of laboratory animals, University of Science and Technology Bannu.

### Plant preparation and extraction

Mature and fresh *T. alexandrinum* whole plants were collected from Bannu district, Pakistan. The taxonomic identification was made by Prof. Abdur Rahman, Department of botany, post graduate college Bannu and voucher specimen was deposited. The plant was dried under shade for 30 days and ground mechanically. The plant powder (800 g) was soaked in 2.5 L of 80% methanol for 7 days at room temperature, with random shaking, filtered through Whatman filter paper No 1. The filtrate was evaporated with the help of a rotary evaporator at 37°C under reduced pressure. The crude *T. alexandrinum* methanolic extract (TAME) was stored at 4°C for further analysis.

### Experimental process and treatment

Thirty male Wistar rats (6 weeks old) were kept at 25°C on a 12 h light/dark cycle, with free access to standard laboratory foodstuff and fresh water. The rats were acclimatized to laboratory conditions for 12 days before designing the experiment. For induction of oxidative stress studies, a 2-week experiment was designed wherein 24 rats were randomly divided into 5 groups as follows: Group (A) was the control, received normal diet and water. Group (B) was injected intraperitoneally (i.p.) with 1 mL CCl_4_/kg b.w. (30% CCl_4_/70% olive oil) after 48 h for 2 weeks. Group (C) was treated with 200 mg/kg b.w. TAME and Group (D) was administered 50 mg/kg b.w. Silymarin orally after 24 h of CCl_4_ treatment while Group (E) was treated with 200 mg/kg b.w. TAME alone for 2 weeks. After 24 h of the last treatment, all the rats were anesthetized and blood was collected to obtain serum by centrifugation at 6000*g* for 15 min. The kidneys were removed and stored at −70°C for further analysis.

### Assessment of urine and serum markers

Before killing rats were kept individually in metabolic cages for 24 h to collect their urine for estimation of renal function tests. Urine samples were assayed using standard diagnostic kits (MediScreen Urine Strips, Orgenics, France). Serum analysis was carried out through standard AMP diagnostic kits (Stattogger Strasse 31b 8045, Graz, Austria).

### Assessment of antioxidant enzymes

For assessment of antioxidant enzymes activity, 100 mg kidneys were homogenized in 4 volume of ice-cold NaH_2_PO_4_ buffer (100 mmol/L, pH 7.4) containing EDTA (1 mmol/L, pH 7.4) for the determination of tissue protein and antioxidant enzymes. Protein concentration was determined by the method of Lowry et al. (1951) using crystalline bovine serum albumin as standard. CAT and POD activities were determined (Chance and Maehly 1955; Manna et al. 2006) with some modifications.

### Glutathione peroxidase assay

GSH-Px activity was measured by using reduced NADPH as a substrate (Mohandas et al. 1984). An extinction coefficient of 6.22 × 10^3^ mol/L^−1^ cm^−1^ at 340 nm was used for calculation.

### Glutathione-*S*-transferase assay

Glutathione-*S*-transferase (GST) activity was determined according to Habig et al. (1974) using CDNB as a substrate. GST was measured at 340 nm using a molar extinction coefficient of 9.6 × 10^3^ mol/L^−1^ cm^−1^.

### Reduced glutathione assay

Reduced glutathione was estimated by the method of Jollow et al. (1974) using 1,2-dithio-bis nitro benzoic acid (DTNB) as substrate. The yellow color developed was read immediately at 412 nm and expressed as *μ*mol GSH/g tissue.

### Estimation of lipid peroxidation assay

Thiobarbituric acid-reactive substances (TBARS) were measured at 535 nm by using 2-thiobarbituric acid (2,6-dihydroxypyrimidine-2-thiol; TBA). An extinction coefficient of 156,000 mol/L^−1^ cm^−1^ was used for calculation according to Iqbal et al. (1996).

### Statistical analysis

Data are presented as mean standard deviation. Computer software (SPSS version 16) was used for statistical analysis. The analysis of variance and post hoc multiple comparison tests were done to estimate the differences among the different groups. The value of *P* < 0.05 was considered significant.

## Results

### Phytochemical analysis

**Figure 1:**
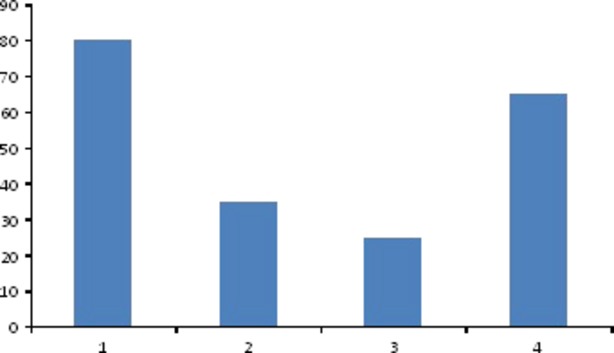
Phytochemical screening of TAME (1: concentration of alkaloids; 2: cumarine; 3: cardiac glycoside; 4: flavonoids).

Phytochemical analysis of TAME revealed the presence of alkaloids, coumarins, cardiac glycoside, and flavonoids as shown in Figure1.

### In vitro antioxidant activity

**Figure 2:**
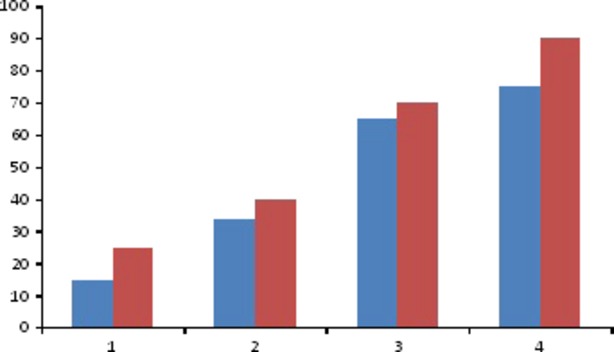
In vitro antioxidant activity of TAME and ascorbic acid (1: 5 *μ*g/mL; 2: 10 *μ*g/mL; 3: 50 *μ*g/mL; 4: 100 *μ*g/mL).

In this part of study the antioxidant potential of TAME was investigated as a part of our efforts to find out pharmacological level of fruit. Results obtained in this context are revealed that scavenging of free radicals is dose dependent as shown in Figure2.

### Effect of TAME on urobilinogen, pH and specific gravity WBCs and RBCs in urine

Protective effects of TAME against CCl_4_ administration abnormality in urobilinogen, pH, specific gravity, white blood cells (WBCs) and red blood cells (RBCs) of urine are shown in given Table1. Intoxication of CCl_4_ significantly (*P* < 0.01) increased the contents of urobilinogen, specific gravity, WBCs, and RBCs while it decreased the pH of urine as compared to the control group. TAME and Silymarin significantly (*P* < 0.01) ameliorated the effects of CCl_4_ analyzed at the urine level.

**Table 1:** Effect of TAME on physical and biochemical parameters in urine of rat.

Treatment	Urobilinogen (mg/dL)	pH	Specific gravity	RBC/*μ*L	WBC/*μ*L
Control	6.0 ± 0.2^2^	7.0 ± 0.8^2^	1.03 ± 0.3^2^	0.0 ± 0.0^2^	20.2 ± 2.9^2^
CCl_4_	7.6 ± 0.3^1^	6.5 ± 0.9^2^	1.22 ± 0.6^1^	10.0 ± 0.07^2^	80.0 ± 7.9^1^
TAME + CCl_4_	6.8 ± 0.4^2^	7.1 ± 0.6^2^	1.01 ± 0.4^2^	2.0 ± 0.06^2^	30.2 ± 3.2^2^
Silymarin + CCl_4_	6.3 ± 0.8^2^	7.0 ± 0.7^2^	1.01 ± 0.9^2^	1.0 ± 0.03^2^	24.4 ± 4.0^2^
TAME alone	5.9 ± 0.5^2^	7.3 ± 0.5^2^	1.06 ± 0.3^2^	0.0 ± 0.00^2^	21.2 ± 1.0^2^

Mean ± SEM (*n* = 6 number). TAME, *Trifolium alexandrinum* methanolic extract.

1 Indicate significance from the control group at *P* < 0.01 probability level.

2 Indicate significance from the CCl_4_ group at *P* < 0.01 probability level.

### Effect of TAME on serum markers

Treatment of CCl_4_ significantly (*P* < 0.01) decreased serum proteins and albumin while increased creatinine, creatinine clearance and bilirubin contents as compared to the control group. Supplementation of Silymarin and TAME significantly (*P* < 0.01) augmented the serum marker as compare to control group (Table2).

**Table 2:** Effect of TAME on serum biochemical parameters of rat.

Treatment	Creatinine clearance	Protein (mg/dL)	Albumin (mg/dL)	Bilirubin (mg/dL)	Creatinine (mg/dL)
Control	5.0 ± 0.2^2^	12.0 ± 0.8^2^	5.0 ± 0.6^2^	0.7 ± 0.09^2^	0.62 ± 0.03^2^
CCl_4_	7.0 ± 0.4^1^	6.1 ± 0.9^1^	3.4 ± 0.9^1^	1.2 ± 0.08^1^	0.84 ± 0.05^1^
TAME + CCl_4_	6.6 ± 0.7^2^	9.7 ± 1.3^2^	4.0 ± 0.7^2^	0.9 ± 0.07^2^	0.70 ± 0.03^2^
Silymarin + CCl_4_	5.9 ± 0.9^2^	10.9 ± 1.2^2^	4.6 ± 0.8^2^	1.0 ± 0.03^2^	0.67 ± 0.07^2^
TAME alone	5.2 ± 0.3^2^	11.7 ± 1.5^2^	4.9 ± 0.7^2^	0.9 ± 0.07^2^	0.63 ± 0.04^2^

Mean ± SEM (*n* = 6 number). TAME, *Trifolium alexandrinum* methanolic extract.

1 Indicate significance from the control group at *P* < 0.01 probability level.

2 Indicate significance from the CCl_4_ group at *P* < 0.01 probability level.

### Effect of TAME on protein and antioxidant enzymes

Changes in kidney protein contents and antioxidant enzymes CAT, POD, GSH-Px and GST are shown in Table3. Administration of CCl_4_ significantly (*P* < 0.01) increased the protein contents while decreased the activities of CAT, POD, GSH-Px and GST to that of control. Treatment of TAME significantly (*P* < 0.01) increased the protein and activities of antioxidant enzymes toward the normal level.

**Table 3:** Effect of TAME on the protein and antioxidant enzymes in kidney of rat.

Treatment	Protein (*μ*g/mg tissue)	CAT (U/min)	GST (nmol min^−1^ mg^−1^ protein)	POD (U/min)	GSH-Px (mol/g tissue)
Control	65.6 ± 5.2^2^	1.5 ± 0.05^2^	188.90 ± 12.4^2^	1.1 ± 0.02^2^	47.4 ± 0.9^2^
CCl_4_	26.3 ± 4.3^1^	0.9 ± 0.02^1^	95.90 ± 13.2^1^	0.5 ± 0.06^1^	23.6 ± 0.7^1^
TAME + CCl_4_	50.0 ± 5.2^2^	1.0 ± 0.06^2^	100.0 ± 8.9^2^	0.8 ± 0.05^2^	33.6 ± 1.2^2^
Silymarin + CCl_4_	63.0 ± 7.1^2^	1.4 ± 0.03^2^	164.0 ± 14.6^2^	1.0 ± 0.04^2^	39.3 ± 2.3^2^
TAME alone	66.2 ± 4.5^2^	1.48 ± 0.07^2^	186.3 ± 13.2^2^	1.3 ± 0.04^2^	49.2 ± 2.3^2^

Mean ± SEM (*n* = 6 number). CAT, catalase; GSH-Px, glutathione peroxidase; GST, glutathione-*S*-transferase; POD, peroxidase; TAME, *Trifolium alexandrinum* methanolic extract.

1 Indicate significance from the control group at *P* < 0.01 probability level.

2 Indicate significance from the CCl_4_ group at *P* < 0.01 probability level.

### Effect of TAME on GSH and TBARS

Treatment of CCl_4_ significantly (*P* < 0.01) decreased GSH while increased TBARS contents comparatively to control (Table4). Co-treatment with Silymarin and TAME showed the ameliorating effects and significantly (*P *< 0.01) reversed TBARS and GSH contents.

**Table 4:** Effect of TAME on GSH and TBARS.

Treatment	GSH (mol/g tissue)	TBARS (nmol/min mg^−1^ protein)
Control	122.4 ± 10.3^2^	1.56 ± 0.03^2^
CCl_4_	47.4 ± 8.9^1^	3.90 ± 0.05^1^
TAME + CCl_4_	80.0 ± 12.4^2^	2.30 ± 0.09^2^
Silymarin + CCl_4_	110.2 ± 9.8^2^	1.86 ± 0.07^2^
TAME alone	118.6 ± 7.3^2^	1.49 ± 0.00^2^

Mean ± SEM (*n* = 6 number). GSH, glutathione; TAME, *Trifolium alexandrinum* methanolic extract; TBARS, thiobarbituric acid-reactive substances.

1 Indicate significance from the control group at *P* < 0.01 probability level.

2 Indicate significance from the CCl_4_ group at *P* < 0.01 probability level.

### Effect of TAME on body weight, kidney, and relative kidney weight

Protective effects of TAME against CCl_4_ administration in rat kidney weight, relative kidney weight are shown in Table5. CCl_4_ significantly altered (*P* < 0.01) the body, kidney and relative kidney weight versus the control group. However, co-treatment of TAME ameliorated the CCl_4_ toxicity and significantly recovered (*P* < 0.01) the body, kidney and relative kidney weight. Ameliorating effects of TAME were observed for these parameters were similar to the effect of Silymarin.

**Table 5:** Effect of TAME on body and kidney weight of rats.

Treatment	Initial b.w. (g)	Final b.w (g)	% increase b.w.	Absolute kidney weight	Relative kidney weight
Control	175.3 ± 11.3^2^	207 ± 10.6^2^	6.85 ± 0.5^2^	1.02 ± 0.08^2^	0.54 ± 0.01^2^
CCl_4_	180.5 ± 10.7^1^	188 ± 9.8^1^	4.44 ± 0.3^1^	1.43 ± 0.05^1^	0.76 ± 0.03^1^
TAME + CCl_4_	185.2 ± 12.5^2^	216 ± 7.9^2^	5.94 ± 0.8^2^	1.2 ± 0.07^2^	0.61 ± 0.04^2^
Silymarin + CCl_4_	180.1 ± 13.3^2^	212 ± 8.9^2^	6.66 ± 0.9^2^	1.0 ± 0.03^2^	0.52 ± 0.02^2^
TAME alone	134.3 ± 12.5^2^	202 ± 3.5^2^	6.87 ± 0.5^2^	1.0 ± 0.07^2^	0.55 ± 0.03^2^

Mean ± SEM (*n* = 6 number). TAME, *Trifolium alexandrinum* methanolic extract.

1 Indicate significance from the control group at *P* < 0.01 probability level.

2 Indicate significance from the CCl_4_ group at *P* < 0.01 probability level.

### Effects of TAME on renal histopatlogy

The sections of control group showed normal histology with normal glomerulus, bowman capsule, distal and proximal convoluted tubules. CCl_4_ treatments in rat cause markedly histological changes and showed tubular and glomerular injuries (Table6). Treatment of TAME to CCl_4_-treated rats markedly recovered the toxic changes near to the control rat.

**Table 6:** Effect of TAME on histopathology.

Treatment	Dilatation	Necrosis	Congestion	Glomerular injuries
Control	−	−	−	−
CCl_4_	++	−/+	−/+	−/+
TAME + CCl_4_	–	–	−	–
Silymarin + CCl_4_	−	−	−	−
TAME alone	−	−	−	−

−, normal; −/+, mild; ++, medium damaged. TAME, *Trifolium alexandrinum* methanolic extract.

## Discussions

In the present investigations the effect of TAME on the antioxidant condition of CCl_4_ intoxicated rats was studied. For this purpose we studied the effects TAME on activity of kidney antioxidant enzymes and lipid peroxidation. Alterations in the antioxidant enzymes status were observed as a result of the CCl_4_-induced oxidative stress which is associated with the elevation of lipid peroxidation and TBARS accumulation in the kidney of rats (Dashti et al. 1989; Behar-Cohen et al. 1996; Khan et al. 2009, 2010a).

The oxidation of lipids in plasma membrane can also generate free radicals that react with polyunsaturated fatty acid of the cell and resulting in lipid per-oxidation. The concentration of TBARS was increasing as a result of lipid peroxidation in the kidney tissues of rats. On the other hand, antioxidant enzymes like CAT and POD, GSH-Px and GST play an important role against oxidative stress. The results showed that the administration of TAME improved the alterations of antioxidant enzymes level and lipid per-oxidation in CCl_4_ intoxicated rats. These results suggest that might act as a suppressor against tissue damage by improvement of the antioxidant enzymes activities. The polyphenolic compounds of *T. alexandrinum* (L) can act as effective free radical scavenger and inhibit lipid per-oxidation by the restoration of antioxidant enzymes activities (Amer et al. 2004). The presence of flavonoids in the methanolic fraction of *T. alexandrinum* might be responsible for the protective effects of oxidative stress induced by CCl_4_ in kidneys of rats (Vardavas et al. 2006; Alpinar et al. 2009).

Results obtained from this study are suggested that TAME possess protective effects against the renal-induced dysfunction. The status of kidney function and acid base balance can be examined by analysis of urine (Trease and Evans 1989). In normal condition urine has no urobilinogen contents. Urobilinogen is considered to be the end product of conjugated bilirubin. The presence of high levels of urobilinogen, creatinine and albumin in urine is the sign of kidney injuries caused by CCl_4_ (Free and Free 1972; Pels et al. 1989). The value of Specific gravity and pH correlates with infections takes place with CCl_4_ intoxication (Ozturk et al. 2003).

Results revealed that CCl_4_ significantly increased urinery protein, RBCs and WBCs showing kidney injuries. High level of proteinurea and haematuria in urine in this experiment showed the CCl_4_ induced nephrotoxicity (Khan et al. 2010a). The present study revealed that oral administration of TAME significantly improved creatinine and urobilinogen.

This study showed that CCl_4_ caused marked defect in renal functions and caused oxidative stress in the kidneys. Serum creatinine, urobilinogen and bilirubin, concentrations were higher in the CCl_4_-treated rats (Ogawa et al. 1992; Adewole et al. 2007). Silymarin and TAME significantly improved and decreased the elevated levels of creatinine and bilirubin. In addition, a reduced level of serum albumin in CCl_4_-treated rats might be indicated due to the injuries in the glomerulus which was restored by TAME and Silymarin. GSH-Px catalyzes the GSH-dependant reduction of hydrogen peroxide (H_2_O_2_) and protects the cells from damage. GSHs show high reactivity toward lipid peroxides. Decrease in GSTs and GSH-Px level due to CCl_4_ toxicity was observed due to lesser availability of GSH. Administration of TAME improved the CCl_4_ toxicity. Similar results were reported by Bhadauria et al. (2008). In the present study, administration of CCl_4_ increased the kidney and relative kidney weight that may be due to the accumulation of proteins in the cells. Supplementation of Silymarin and TAME reversed the changes to a normal level.

Hepatohistology of CCl_4_ intoxicated rats revealed immense fatty changes, glomerular dilation and tubular injuries which were markedly diminished by induction of TAME. Our study revealed a similar investigation which is in agreement with earlier findings while evaluating the medicinal activity of plants against CCl_4_-stimulated nephrotoxicity in rats (Khan et al. 2009, 2010a, 2010b).

## Conclusion

Results showed that TAME contributes its protective role by decreasing the action of CCl_4_ at various metabolic cycles. This study authenticated the scientific confirmation in favour of its pharmacological use in renal disorders in folk medicine which might be due to the presence of bioactive constituents present in TAME.
